# Design of fusion enzymes for biocatalytic applications in aqueous and non-aqueous media

**DOI:** 10.3389/fbioe.2022.944226

**Published:** 2022-07-22

**Authors:** Yu Ma, Ningning Zhang, Guillem Vernet, Selin Kara

**Affiliations:** ^1^ Biocatalysis and Bioprocessing Group, Department of Biological and Chemical Engineering, Aarhus University, Aarhus, Denmark; ^2^ Institute of Technical Chemistry, Leibniz University Hannover, Hannover, Germany

**Keywords:** fusion enzymes, biocatalytic cascades, oxidoreductases, fusion linkers, aqueous and non-aqueous media

## Abstract

Biocatalytic cascades play a fundamental role in sustainable chemical synthesis. Fusion enzymes are one of the powerful toolboxes to enable the tailored combination of multiple enzymes for efficient cooperative cascades. Especially, this approach offers a substantial potential for the practical application of cofactor-dependent oxidoreductases by forming cofactor self-sufficient cascades. Adequate cofactor recycling while keeping the oxidized/reduced cofactor in a confined microenvironment benefits from the fusion fashion and makes the use of oxidoreductases in harsh non-aqueous media practical. In this mini-review, we have summarized the application of various fusion enzymes in aqueous and non-aqueous media with a focus on the discussion of linker design within oxidoreductases. The design and properties of the reported linkers have been reviewed in detail. Besides, the substrate loadings in these studies have been listed to showcase one of the key limitations (low solubility of hydrophobic substrates) of aqueous biocatalysis when it comes to efficiency and economic feasibility. Therefore, a straightforward strategy of applying non-aqueous media has been briefly discussed while the potential of using the fusion oxidoreductase of interest in organic media was highlighted.

## 1 Introduction

Biocatalytic cascades have been widely explored to mimic natural biosynthetic routes to produce high value-added chemicals (Muschiol et al., 2015; France et al., 2017; Huffman et al., 2019; Walsh and Moore, 2019; Zhou et al., 2021; Benítez-Mateos et al., 2022). In this context, the spatial organization of multi-enzymes plays a pivotal role in surmounting barriers between different enzyme classes, averting the mutual inhibition, limiting the long-range diffusion of intermediates, and enhancing the reaction efficiency (Quin et al., 2017). Up to now, a variety of enzyme co-localization strategies have been developed including 1) enzyme fusion (Elleuche, 2015; Aalbers and Fraaije, 2019), 2) co-immobilization (attachment on a carrier or encapsulation in a matrix) (Ren et al., 2019; Xu et al., 2020), and 3) scaffolding (Kuchler et al., 2016; Ellis et al., 2019). In particular, the direct fusion of enzymes has emerged as a fascinating toolbox in its own right (Quin et al., 2017; Rabe et al., 2017; Aalbers and Fraaije, 2019). It brings multiple enzymes in close proximity to form a single multifunctional catalyst through genetic fusion (Aalbers and Fraaije, 2017) or covalent bonds between proteins formed post-transcriptionally (Keeble and Howarth, 2020). By enzyme fusion, the sequential channeling of substrates between enzyme active sites can be easily achieved with a high degree of controllability (Huang et al., 2001; Wilding et al., 2018). Consequently, the delicately tethered enzymes gain many benefits, such as improved stability and catalytic efficiency, as well as enhanced expression and ease of production as a single construct.

**SCHEME 1:**
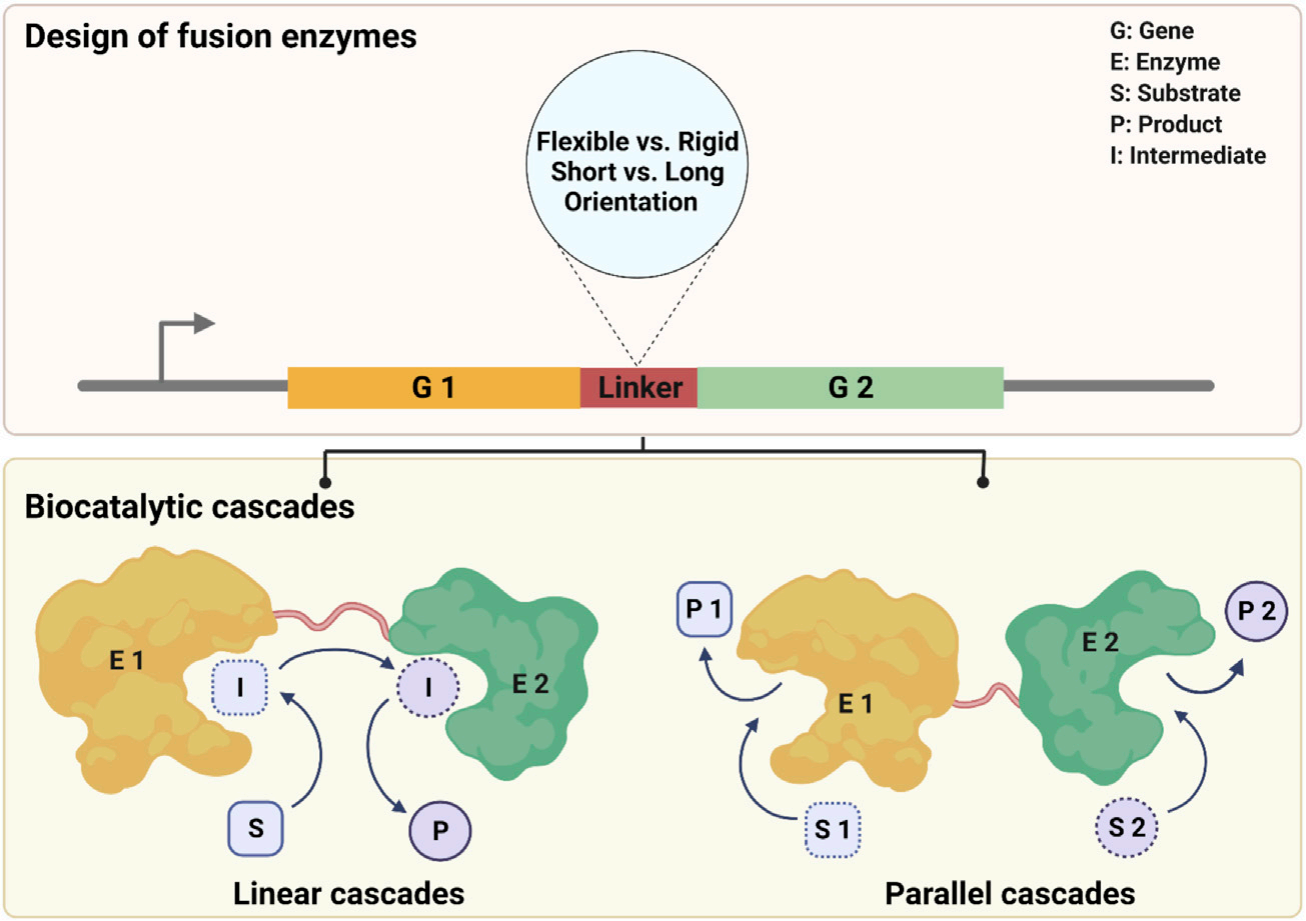
Design of fusion enzymes for two biocatalytic cascade processes.

Fusion approaches have been well-illustrated with many types of cofactor-dependent enzymes including the combination of cytochrome P450 with their redox partners (Munro et al., 2007; Bakkes et al., 2017; Kokkonen et al., 2019; Belsare et al., 2020; Kokorin et al., 2021; Kokorin and Urlacher, 2022), and Baeyer-Villiger monooxygenases (BVMOs) with alcohol dehydrogenases and transaminases (Torres Pazmiño et al., 2008; Peters et al., 2017; Aalbers and Fraaije, 2019) (Scheme 1). These studies have largely demonstrated the practicability and effectiveness of the fusion approach. However, there are still some limitations to overcome in order to make it practicable. The trial-and-error in the design of linkers and time-consuming molecular experiments can lead to a huge workload. Furthermore, most of these proofs of concept were explored at low substrate loadings usually in μM ranges, which lags far behind practical applications at a technical scale.

Some enzymes are of high interest for their use in cascades for the biocatalytic production of valuable chemicals. In particular, cyclohexanone monooxygenase (CHMO) has been extensively investigated for the synthesis of ɛ-caprolactone, an important precursor for polymer synthesis (Schmidt et al., 2015; Scherkus et al., 2016; Engel et al., 2019a). Generally, most cascades have been developed by coupling various alcohol dehydrogenases (ADHs) (Bornadel et al., 2015; Schmidt et al., 2015; Scherkus et al., 2016; Scherkus et al., 2017; Engel et al., 2019b; de Gonzalo and Alcántara, 2021) as well as newly reported thioredoxin/thioredoxin reductases pairs (Zhang et al., 2022) with CHMO to form redox-neutral self-sufficient systems. Despite these advances, in most one-pot systems, substrate concentrations have been used up to 100 mM given the strong substrate and product inhibition. By harnessing the fusion approach not only the inhibition caused by substrates and intermediates could be relieved but also the transport distance of cofactors between active sites can be shortened to avoid the degradation of nicotinamide cofactors in non-aqueous media (Huang et al., 2019). This has inspired the design and generation of various fusions of CHMO with its cofactor regenerating enzymes as effective catalysts for cascades, demonstrating the practicality of fusions for this type of enzyme (Aalbers and Fraaije, 2017).

Enzymes have naturally evolved to function optimally in aqueous media. Accordingly, most enzyme-catalyzed transformations take place in buffer systems to maintain enzymatic stability and activity. However, for industrial applications that mostly use hydrophobic non-natural substrates, this can be a key obstacle (Holtmann and Hollmann, 2022). In most studies, the concentrations of poorly water-soluble reagents are often adjusted to the millimolar range. The ‘diluted’ biocatalysis with low substrate loadings is both poorly economical and unsustainable (Hollmann et al., 2021). Using non-aqueous media with the presence of co-solvents can effectively enable high substrate loadings required by the industrial scale-up and commercialization (Dominguez de Maria and Hollmann, 2015; Illanes, 2016). Biocatalysis in non-aqueous media has seen a big rise since the pioneering work of Klibanov (1989) on enzyme catalysis in organic solvents (Zaks and Klibanov, 1988), which mainly focused on lipases with distinctive stability (Kumar et al., 2016). However, applying redox biocatalysis in non-aqueous media confronts several major concerns, e.g., enzyme stability and cofactor recycling (Kara et al., 2013; Huang et al., 2018; Vidal et al., 2018). A recent study proved the feasibility of using a fused type II flavin-containing monooxygenase (FMO-E) and horse liver alcohol dehydrogenase (HLADH) in organic media (Huang et al., 2019). Benefits can be promisingly envisioned by applying these fusions for cascades with high substrate loadings in non-aqueous media (Holtmann and Hollmann, 2022). Inspiringly, many enzymes of interest especially the above-mentioned CHMO can be optimized by a similar way for the cascades toward an industrial application.

Here we present recent advances in the design and application of fusion enzymes, which cover not only the traditional aqueous systems but also non-aqueous media (e.g., organic solvents, and deep eutectic solvents). In detail, the development and application of various fusion enzymes in an aqueous environment (Section 1) and some recent examples especially about oxidoreductases in non-aqueous media (Section 2) have been outlined. Meanwhile, the linker design regarding classification, flexibility and rigidity, length, and orientation is discussed in both sections. In particular, the potential of using fusion enzymes for redox cascades with high substrate loadings in pursuit of satisfactory space-time-yields is highlighted.

## 2 Fusion enzymes in aqueous media

Most fused enzymes are constructed by genetic fusion, which requires a linker peptide to connect target proteins. A linker peptide is a segment of the polypeptide, consisting of several or hundreds of amino acids in length (Arai, 2021). The presence of linkers allows for the separation of two enzymes and thus avoids mutual interference during the folding and catalytic processes. There are at least two factors to consider when designing an enzyme fusion: 1) which type of linker to use and 2) in which order proteins should be placed. In the first aspect, the composition and length of a linker are two determining factors of its physicochemical properties regarding flexibility vs. rigidity and hydrophilicity vs. hydrophobicity. This can highly affect the spatial distribution of fused subunits. According to the characteristics of linkers, they are generally classified into two types: 1) flexible linker peptides and 2) linker peptides that can form α-helices (Chen et al., 2013). As a simple summary, Table 1 lists the enzyme pairs in fused or non-fused forms and the used linkers of mainly oxidoreductases described in this mini-review. All examples are explained in more detail in the entries.

**TABLE 1:** List of fused and non-fused oxidoreductases, and their biocatalytic applications with varying substrate concentrations in various aqueous and non-aqueous media.

Entry	Enzyme pairs	Fusion name	Linker	Application of enzyme pairs	Substrate concentration (mM)	Reaction media	Organic solvent (vol%)	References
1	ADH, BVMO	CHMO-ADHA	L1: (13) SSGGSGGSGGSAG	cascade reaction, cyclic alcohol to lactone	0.25, 10	water	0	Aalbers and Fraaije, (2017)
		CHMO-ADHM						
		ADH-CHMO						
2	ADH, BVMO	FDH-CHMO	L1: (6) SGSAAG L2: (6) SRSAAG	NADPH-recycling system	5	water, MTBE, DES (choline chloride and glycerol)	0, 10, 40, 20	Mourelle-Insua et al. (2019)
		GDH-CHMO						
		PTDH-CHMO						
		FDH-ADH						
		GDH-ADH						
		PTDH-ADH						
3	ADH, BVMO	ADH-Gly-BVMO	L1: (12) SGGSGGSGGSAG L2: (30) SASNCLIGLFLNDQELKKKAKVYDKIAKDV L3: none	cascade reaction, alcohol to ester	0.2	water	0	Jeon et al. (2015)
		ADH-FOM-BVMO						
		ADH-BVMO						
4	Ene reductase, BVMO	XenB-CHMO	L1: (13) SSGGSGGSGGSAG L2: (12) SSATGSATGSAG L3: (1) W	cascade reaction, unsaturated cyclic alcohols to chiral lactones	3	water	0	Peters et al. (2017)
5	PTDH, P450	BF2	L1: (6) SGGGGS L2: (6) EPPPPK L3: (24) (SGGGGS) × 4	NADPH-recycling system	0.2	water	0	Kokorin et al. (2021)
		F2B						
		F2B-P1						
		F2B-G1						
		F2B-G4						
6	PTDH, P450	pCRE2-P450-BM3	L1: (6) SRSAAG	NADPH-recycling system	0.1	water	0	Beyer et al. (2018)
7	Styrene monooxygenase (StyA), Flavin reductase (StyB)	Fus-SMO	L1: (30) ASGGGGSGGGGSGGGGSGGGGSGGGGSGAS L2: (20) (GGGGS) × 4	electron transfer for epoxidation of styrene	0.5	water	0	Corrado et al. (2018)
8	P450, Alcohol oxidase	OleTJE-AldO	L1: (18) GSGLEVLFQGPGSGGGGS L2: (45) A (EAAAK) × 4-LEA-(EAAAK) × 4A	hydrogen peroxide supply for decarboxylation reaction	0.5–10	water	0	Matthews et al. (2017)
9	Formate dehydrogenase	FDH-AzoRo	L1: (30) His Tag × 10	NAD^+^ regeneration	0.025	water	0	Ngo et al. (2022)
10	ADH, aminotransferase	ADH-AT	L1: PAS linker: (20) ASPAAPAPASPAAPAPSAPA L2: (40) PAS × 2 L3: (60) PAS × 3	cascade reaction, alcohol to amine, stabilization through linker	300	water	0	Lerchner et al. (2016)
11	Formate dehydrogenase, Leucine dehydrogenase	FDH-LeuDH	L1: none L2: (5) EAAAK L3: (10) (EAAAK) × 2 L4: (15) (EAAAK) × 3 L5: (5) GGGGS L6: (10) (GGGGS) × 2 L7: (15) (GGGGS) × 3	L-tert leucine biotransformation	4.5	water	0	Zhang et al. (2017)
12	P450 BM3	BM3-ADH	L1: none L2: (10) (GGGGS) × 2 L3: (9) A × 9 L4: (10) (EAAAK) × 2	NADPH-recycling system	0.2, 0.5, 10	water	0	Kokorin and Urlacher, (2022)
		ADH-BM3						
13	Flavin-dependent halogenase, flavin reductase	FH-FR	L1: (10) PSPSTDQSPS L2: (16) VLHRHQPVTIGEPAAR L3: (22) VLHRHQPVSPIHSRTIGEPAAR	electron transfer for halogenation	0.5	water	0	Andorfer et al. (2017)
14	CHMO, ADH, CAL-A	No fused	—	—	20, 100	water	0	Schmidt et al. (2015)
15	CHMO	No fused	—	—	3.4–11	water	0	Romero et al. (2016)
16	CHMO, ADH, CAL-B	No fused	—	—	1–25	water	0	Scherkus et al. (2016)
17	P450, ADH	No fused	—	—	2, 20	water	0	Tavanti et al. (2017)
18	PTDH, BVMO	PockeMO-PTDH	L1: (6) SRSAAG	NADPH-recycling system	0.2–0.8	water, dioxane	10	Furst et al. (2017)
		CPDMO-PTDH						
		CHMO-PTDH						
19	CHMO, ADH, CAL-B	No fused	—	—	20	water	0	Scherkus et al. (2017)
20	CHMO, ADH, CAL-B	No fused	—	—	40–100	water	0	Wedde et al. (2017)
21	CHMO, ADH	No fused	—	—	0, 100	water	0	Engel et al. (2019b)
22	CHMO, GDH	No fused	—	—	10, 140	water, methanol	10	Delgove et al. (2019)
23	CHMO, GDH	No fused	—	—	30, 240	water, methanol	1.25, 10	Solé et al. (2019)
24	FMO, ADH	FMO-ADH	L1: (6) SGSAAG	NADPH-recycling system	10–20	microaqueous	95	Huang et al. (2019)
25	PSMO, FDH	No fused	—	—	10	water, methanol	10	Zhu et al. (2022)
26	CHMO, FDH	No fused	—	—	5	water, methanol	10	Zhu et al. (2022)

CHMO, Cyclohexanone monooxygenase; ADH, Alcohol dehydrogenase; PTDH, Phosphite dehydrogenase; FDH, Formate dehydrogenase; GDH, Glucose dehydrogenase; PockeMO, Polycyclic ketone monooxygenase; CPDMO, Pseudomonad cyclopentadecanone monooxygenase; MTBE, Methyl tert-butyl ether; DES, Deep eutectic solvent.

The composition is a determinant factor for the flexibility or rigidity of linkers. Flexible linkers are glycine-rich and can produce a disordered loop, which usually could improve protein solubility and provide flexibility for catalysis domain separation (Arai et al., 2001). The flexible linker peptide does not interfere with the folding domain of the protein, thus theoretically allowing for natural folding and other conformational movements (Reddy Chichili et al., 2013). This type of linker has been widely used in biocatalysis with relative success, such as cytochrome P450 fusions (Matthews et al., 2017; Beyer et al., 2018; Kokorin et al., 2021; Kokorin and Urlacher, 2022), flavin reductase (FR) fusions (Andorfer et al., 2017; Corrado et al., 2018), formate dehydrogenase (FDH) fusions (Zhang et al., 2017), and Baeyer-Villiger Monooxygenases (BVMOs) fusions (entries 1–13, Table 1). In detail, Fraaije and coworkers reported a fusion of an ADH from *Thermoanaerobacter brockii* (*Tb*ADH) and a CHMO from *Thermocrispum municipal* (*Tm*CHMO) with a glycine-rich linker, which was used in a linear cascade fashion to synthesize ε-caprolactone (entry 1, Table 1) (Aalbers and Fraaije, 2017). They found the fused *Tm*CHMO exhibited around two-fold higher oxygenation activity compared to the individual protein. The obtained fusion achieved 99% conversion using 200 mM substrate (substrate-feeding applied) and gave a turnover number (TON) of >13,000 (Aalbers and Fraaije, 2017). Glycine-rich peptide linkers are structurally flexible and thus hardly restrict the natural movement of enzymes, which to a high extent leads to a more favourable performance of fusion enzymes compared to that with rigid linkers. There is another example of such enzyme that *Tm*CHMO was fused with three different cofactor regeneration enzymes using short flexible linkers. Therein all fusion enzymes resulted in good soluble expression and excellent conversions (entry 2, Table 1) (Mourelle-Insua et al., 2019). Not only for isolated enzyme fusions, but also the biotransformation activity of recombinant cells containing overexpressed fusion enzymes was markedly influenced by the type of fusion linkers. Therein, it turned out that flexible linkers allowed for higher conversions than rigid α-helix linkers (entry 3, Table 1) (Jeon et al., 2015). In general, other studies have shown some similar effects by using flexible linkers, reaching higher conversions (Peters et al., 2017; Kokorin et al., 2021), improving catalytic activities (Beyer et al., 2017; Beyer et al., 2018; Corrado et al., 2018), and yielding higher productions (Peters et al., 2017) (entries 4–7, Table 1). Therefore, flexible glycine-rich linkers are safe options to try when it comes to the preliminary linker design.

The inclusion of helix-associated amino acids such as alanine and lysine enables the introduction of stiff tethers in glycine-rich linkers, providing the advantage of being resistant to proteolysis and the well-controlled domain separation (Chen et al., 2013). In some research, it has been studied that fusions with rigid peptide linkers exhibited better activities than that with flexible linkers due to the effective separation of protein moieties (Zhang et al., 2017; Huang et al., 2021). For example, the α-helix-based rigid linker between alditol oxidase (AldO) and cytochrome P450 OleTJE (CYP152L1) highly contributed to the improved decarboxylation of myristic acid in the presence of peroxide (H_2_O_2_) when compared to equal amounts of isolated OleTJE and AldO (entry 8, Table 1). The authors also mentioned the enhanced activity may be attributed to the more efficient channeling of H_2_O_2_ between enzyme active sites within the proximity of these domains (Matthews et al., 2017).

Despite the potential of rigid linkers, flexible linkers have received more attention than the research done so far on rigid linkers. Overall, various studies have described glycine-rich linkers are beneficial by enhancing flexibility between the two partners, which could provide degrees of freedom for proper folding and conformational changes (Chen et al., 2013). When most studies have focused on the use of flexible or rigid linkers, there is an interesting study that reported a fusion of FDH from *Candida boidinii* and azoreductase from *Rhodococcus opacus 1CP* (AzoRo) with His 10-tag as the linker (Ngo et al., 2022). Due to its high affinity for nickel-containing resins, histidine (His) is often designed as a tag for affinity purification of recombinant proteins. Since most recombinant proteins usually have His-tags at the N or C-terminal, His-tags are used to combine the two proteins with the expectation that it can have multiple biological functions of proteins purification and fusion linkers at the same time. The result showed using His-tag as a linker is achievable, but it might affect the solubility of the fusion protein (entry 9, Table 1). Evidently, each of these linkers has its own pros and cons, and the application will depend on the specific reaction to be achieved.

Besides the composition of linkers, the length of linkers has recently been found to have a pronounced effect on the properties of fusion enzymes. In one case, the effect of the length of a glycine-rich linker with 15 amino acids as the basic linker on the biocatalytic properties of *Tb*ADH-*Tm*CHMO fusion was investigated by evaluating 14 lengths (Gran-Scheuch et al., 2021). All variants exhibited a high expression level but varying activities. The fusions with linker lengths of 10, 12, and 15 amino acids, showed a slight increase in *k*_obs_ for both activities while the fusions with 2, 3, 6, 7, 13, and 14 amino acid linkers resulted in the highest TONs (Gran-Scheuch et al., 2021). Despite that, no clear correlation between linker length and the catalytic performance of fusions was established within limited studies. Nevertheless, the length of linkers can be adjusted to make space for proper folding of both enzymes, which consequently affects the expression and activity of fusions. For example, three linkers consisting of repeated PAS sequences (20, 40, and 60 amino acids) (entry 10, Table 1) were used in the fusion of an ADH with an aminotransferase (AT) to synthesize amines from alcohols. Therein, they found specific effects for each linker, from short to long: PAS20 achieved two-fold higher conversion compared to the individual enzymes; PAS40 showed the highest activity while PAS60 resulted in the highest soluble expression (Lerchner et al., 2016). Likewise, in another study using NHase as a subunit, proper longer linkers resulted in higher stability while overlong linkers had a negative effect on the activity and expression of NHase. (Guo et al., 2021). Based on current studies, there is still no clear consensus on whether longer or shorter linkers are better. Therefore, it is necessary to design and evaluate different lengths of linkers in a certain case, which, obviously, can be time-consuming. For this reason, a recent study reported a three-step process in straightforward PCR that utilized reiterative primer design, PCR-mediated linker library generation, and restriction enzyme-free cloning methods to generate linker libraries. The authors stated it to be applicable for most fusion constructs (Norris and Hughes, 2018).

In addition to the composition and length of linkers, the order of fused moieties is also critical for the catalytic performance of fusions. An in-depth study of loops and linkers illustrated that linkers are not just ‘connectors’ but have a significant impact on the microenvironment and orientation of fusions (Huang et al., 2021). The order of protein sequences (N and C-terminal orientation) can influence the correct folding, oligomerization state, stability, and activity of the fusion constructs (Lai et al., 2015). Given that, the gene order for a fusion enzyme was exemplarily optimized by using simulations (Lai et al., 2015; Papaleo et al., 2016). Zhang et al. (2017) predicted the orientation of the cofactor binding domain of leucine dehydrogenase (LeuDH) and formate dehydrogenase (FDH) by structural modelling approach with an online server to ensure the favorable orientation of active sites in the fusion enzyme complex (entry 11, Table 1). This simulation revealed that fusing the C-terminus of FDH with the N-terminus of LeuDH formed a favorable face-to-face active cleft orientation. This would promote the formation of intramolecular tunnelling and accelerate the cofactor channel between FDH and LeuDH. However, such a result could not be obtained in the other direction (Zhang et al., 2017). In the case of BVMOs, changing the order of the same linker led to a significant increase in ADH activity: *Tb*ADH-*Tm*CHMO showed higher *k*_cat_ than *Tm*CHMO-*Tb*ADH (Aalbers and Fraaije, 2017). While in another study of P450, there is no difference (entry 12, Table 1) (Kokorin and Urlacher, 2022). In general, the orientation between the two enzymes can have a significant impact on the efficiency of the reaction. Although, with the assistance of computational simulations or using linker databases, the orientation between enzymes can be designed in a more rational way. However, in practice, this is still difficult to control (Lai et al., 2015).

In addition to genetic fusion, post-transcriptional interactions between tags have become popular to bring multiple enzymes in close proximity to form a single multifunctional catalyst (Keeble and Howarth, 2020). SpyTag/SpyCatcher is one such protein coupling approach and it is by far the most used tag. Schoene et al. (2014) reported that locking the termini (often the most flexible part of a protein) together through SpyTag/SpyCatcher altered the enzyme robustness. The thermal and proteolytic stability of β-lactamase has been improved significantly. Another proven way to enhance stability and performance was to encapsulate enzymes with SpyTag/SpyCatcher in protein cages (Mittmann et al., 2022). For example, Pamela and coworkers encapsulated two enzymes via SpyTag/SpyCatcher for the biosynthesis of indigo, enhancing intracellular indigo production and increasing the stability by 90% (Giessen and Silver, 2016). SpyTag/SpyCatcher was shown to facilitate substrate recruitment, thus improving enzyme performance (Wang et al., 2017). Moreover, Spy technology has increased resilience, promoted substrate channeling, and assembled hydrogels for continuous flow biocatalysis (Keeble and Howarth, 2020). Based on these studies, we can see that using a combination of these tags could contribute to a better enzymic performance, especially improving the stability of enzymes. It will be exciting to see how the Spy toolbox develops in the field of biocatalysis in the future.

## 3 Oxidoreductases in organic media and perspectives

As aforementioned, the use of fusion proteins is mostly documented for aqueous media. However, the use of water as a “green” solvent has been intensely debated within the biocatalysis field, which made it clear that the impact of contaminated water surely needs to be quantified when assessing the greenness of an enzymatic process (Ni et al., 2014). For this reason, water has been included in the recently modified E-factor to emphasize a fair comparison between the use of water and other solvents (Tieves et al., 2019). When using water as reaction media for chemical synthesis, not only wastewater but also several other limitations should be considered, such as 1) lower substrate solubility, 2) laborious downstream processing, 3) unwanted water-related side reactions, 4) enzyme inhibition, and 5) microbial contamination. Given these problems especially the limited substrate loadings, there is a high interest in the use of non-aqueous media for enzymatic catalysis (Osterberg et al., 2015; Rosinha Grundtvig et al., 2018; Bollinger et al., 2020). In this context, organic solvents are often widely used in most synthetic processes, especially in the pharmaceutical industry (Caron et al., 2006; van Schie et al., 2021) and can be seen as a possible improvement for different biocatalytic systems.

Research on biocatalysis in organic media has focused on hydrolases (EC 3) mainly lipases some of which have been applied at technical scales. Although oxidoreductases-mediated selective oxidation is considered one of the most important transformations in organic chemistry, studies on oxidoreductases (EC 1) are still limited (Hollmann et al., 2011; Ringborg et al., 2017; Dong et al., 2018). Between 2000 and 2015, 68% of the patents covering biocatalytic applications were based on oxidoreductases, despite the fact that they account for “only” one-third approximately of all the known enzymes (Drenth et al., 2021). The limited use of oxidoreductase at technical scales in organic media is not only due to the instability of redox enzymes *per se* but also the fact that up to 50% approximately of known oxidoreductases are cofactor-dependent [NAD(P)H]. Given the expensive and unstable nature of NAD(P)H, their regeneration and re-utilization are often necessary for the economic feasibility on an industrial scale (Velasco-Lozano et al., 2017; Selles Vidal et al., 2018). In this case, enzyme-coupled cofactor regeneration approaches have emerged as powerful tools to meet this demand. In particular, dehydrogenase-promoted *in situ* cofactor regeneration in whole cells (so-called “designer cells”) (Groger et al., 2006) and *in vitro* multi-enzymatic cascades have been widely reported in aqueous media. In the case of cascade reactions, the distance between enzymes’ active sites has a significant effect. Fusing enzymes or co-immobilizing enzymes are some of the different approaches that can be used to shorten the transport distance of reaction intermediates between active sites while increasing the enzyme stability, jointly contributing to the improved efficiency of cofactor regeneration (Hollmann et al., 2018; Zhu et al., 2022).

Baeyer-Villiger monooxygenases (BVMOs) can oxidize ketones to furnish value-added esters and lactones, which rely on both NADPH and molecular oxygen (Schmidt et al., 2015; Scherkus et al., 2016). One preparative application of BVMO overexpressed in whole cells has been demonstrated in an *in situ* substrate feeding and product removal process by using adsorbent resin within a bubble column reactor (Hilker et al., 2004a; Hilker et al., 2004b; Hilker et al., 2005). This set-up has been scaled up to the kilogram level in a 50 L reactor. Therein, it is concluded that overcoming the oxygen limitation could afford higher productivity. Given the use of organic solvents offers magnitudes higher oxygen solubility than water (Ramesh et al., 2016), applying BVMOs in organic media to achieve oxygen limitation removal is appealing. Furthermore, Sato et al. (2014) reported that the oxygen solubility increases with larger alkyl chains of alcohol solvents while with decreased alkyl chains of alkane solvents, which provided a basic guideline for solvent selection.

Various multi-enzymatic cascades have been designed and established involving oxidoreductases mostly in water and water-organic mixtures to produce bulk and fine chemicals especially active pharmaceutical ingredients (APIs). Some of representative studies have been summarized in Table 1 which has a focus on cyclohexanone monooxygenases and widely used cofactor regeneration enzymes ADHs. These studies demonstrated the screening, the characterization including mutation and fusion (entries 1, 18, 2, and 24, Table 1) for more stable enzymes, and the application of these enzymes in different reaction conditions and set-ups. In addition, the optimization of an oxidative process by using air or pure oxygen was illustrated. All these studies were performed with a broad range of substrate concentrations and various enzymes for the cofactor regeneration (14–17, 19–22, 25, and 26, Table 1). A successful scale-up of a sequential cascade reaction has been reported by adding the two enzymes separately, optimizing the dosing factor, and increasing the reaction volume up to 100 L in a 200 L-reactor (entry 23, Table 1) (Solé et al., 2019). Despite these advances, there is still room for further improvement especially concerning the cofactor recycling, substrate loadings, oxygen supply, and enzyme stability. This is where enzyme fusions in organic media can fit in and provide counterpart solutions.

To the best of our knowledge, there has been only one study on the use of fused oxygenating enzymes in low-water media (micro-aqueous media) so far. Huang et al. (2019) reported the use of fused type II flavin-containing monooxygenase (FMO-E) and horse liver alcohol dehydrogenase (HLADH) by using a flexible linker in micro-aqueous media (5 vol% aqueous buffer in organic solvent) for the synthesis of γ-butyrolactone. It was reported that the enzymes’ tolerance toward organic solvents could be transmitted when enzymes are fused. Depending on the art of combination, different stabilities can be obtained. For instance, Mourelle-Insua et al. (2019) reported that *Bs*FDH-*Tm*CHMO fusion achieved around 10% conversion in 1 vol% 1,4-dioxane, while fusing *Ps*PTDH with *Tm*CHMO by using two different short-flexible linkers led to full conversion. These examples demonstrate the great potential of applying fusion enzymes in organic media. However, it is undoubtable that the rational design of fusion enzymes with suitable linkers and the application of fused enzymes for biocatalytic cascades in non-aqueous media still remains complex and challenging. Moreover, further research is urgently needed to bridge the gap between laboratory-level study and the application under industrially relevant conditions.

## 4 Conclusion and outlook

Overall, the development of fusion enzymes has highly facilitated the design and application of enzymatic cascades to produce valuable compounds in a more efficient manner. Many gains have been firmly demonstrated. The fusion approach enables the combination of target functional domains to easily modularize these multifunctional catalysts for custom applications. For cofactor-dependent enzymes, especially oxidoreductases, fusions have been used to provide efficient cofactor regeneration by shortening diffusion pathways and stabilizing unstable cofactors both in whole cells and *in vitro* using enzyme systems. Not only the improved catalytic performance but also the enhanced co-expression is achievable by optimizing fusion linkers. Given complicated structure-function correlations, rational and efficient design of linkers has so far remained a challenge. However, it is becoming easier with the help of *in-silico* modeling and the establishment of linker database libraries. Along this path, more and more studies have emerged, some of which have been outlined here to provide a general overview. In particular, fusions of oxidoreductase have been highlighted due to high interest in them as well as their infinite potential for chemical synthesis. Benefiting from the fusion mode, the applications of cofactor-dependent oxidoreductases not only in aqueous media but also in non-aqueous media can be realized, expanding the biocatalytic toolbox for sustainable industrial chemistry.
